# A Japanese case of chronic lymphocytic leukemia with t (1;6)

**DOI:** 10.1186/2162-3619-1-28

**Published:** 2012-09-12

**Authors:** Kayo Harada, Kazuhiko Ikeda, Hayato Matsumoto, Miki Furukawa, Hiroshi Takahashi, Hiroshi Ohkawara, Hideyoshi Noji, Kazuhiro Tasaki, Masafumi Abe, Kazuei Ogawa, Yasuchika Takeishi

**Affiliations:** 1 Department of Cardiology and Hematology, Fukushima Medical University, 1 Hikariga-oka, Fukushima, Fukushima, 960-1295, Japan; 2 Department of Pathology and Diagnostic Pathology, Fukushima Medical University, Fukushima, Japan

**Keywords:** CLL, T(1;6), Aggressive clinical course

## Abstract

Chronic lymphocytic leukemia (CLL) rarely exhibits an aggressive clinical course and its patients often have chromosomal deletions or additions. Furthermore, reciprocal translocations are barely observed in CLL. There have only been a few reports of CLL with t(1;6), and here we report the first Asian case of CLL with reciprocal translocation t(1;6). Since our case and previously reported CLL patients with t(1;6) consistently showed aggressive clinical course, t(1;6) may define a distinct type of CLL.

## Introduction

Chronic lymphocytic leukemia (CLL) is a mature B-cell neoplasm characterized by increased abnormal non-large CD5^+^CD20^+^CD23^+^ B cells in peripheral blood, bone marrow, and lymphoid organs [1,2]. CLL usually exhibits an indolent clinical course, but sometimes shows aggressive clinical course, including Richter’s transformation with poor outcome. Poor prognosis in CLL is associated with expression of *CD38* or *zeta-chain associated protein-70* (*ZAP-70*), mutations and/or expression of *tumor protein p53* (*TP53*), and abnormal cytogenetics [3]. The majority of patients with CLL have chromosomal deletions or additions, but chromosomal translocations are rare, and if present, they are usually associated with the loci of *immunoglobulin* (*IG*) genes, 14q32, 2p13, or 22q11 [4]. Also, information on the roles of these translocations in clinical outcome or pathogenesis is extremely limited in CLL.

There are only a few reports of translocation t(1;6) involving chromosome 6p25 ~ 23 in patients with CLL [5-9]. This type of translocation was observed in approximately 0.5% of patients with CLL in the Belgian databases [5]. These cases showed an aggressive clinical course. Furthermore, when treated with purine analogues, patients often develop Richter’s transformation. It has been suggested that a regulator of B-cell differentiation, *interferon regulatory factor 4* (*IRF4*) gene on 6p25, correlates with the pathogenesis of CLL cases with t (1;6) [5].

Here, we report the first Asian case of CLL with translocation t(1;6) where CLL cells expressed IRF4. The patient showed an aggressive clinical course and was successfully treated with conventional chemotherapy without purine analogue.

### Case report

**Figure 1:**
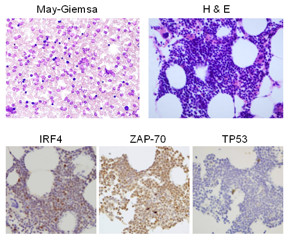
** Histologic findings of bone marrow.** May-Giemsa, H&E, and immunohistochemistrical stainings with indicated antibodies (x400) are shown.

**Figure 2:**
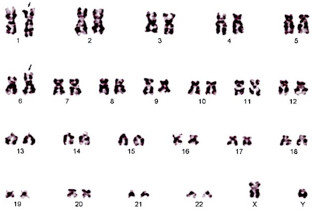
The G-banding shows 46,XY,t(1;6)(p34.1;p23).

A 56-year-old Japanese male, who had taken medical laboratory examinations every half-year, was first found to have a slightly elevated peripheral white blood cell count (12 x 10^9^/L) in July 2010, while showing no other abnormal findings on blood examination and systemic positron emission tomography using ^18^ F-fluorodeoxy glucose. However, only 2 months later he noticed a systemic swelling of lymph nodes, which further rapidly enlarged, and he developed a high body temperature and night sweat. He had a supraclavicular lymph node biopsy in November 2010, and was considered to have advanced B-cell lymphoma because CD20^+^ lymphoid cells proliferated. Based on this finding, he was referred to our department for the purpose of chemotherapy. On admission, he had mild splenomegaly and leukocytosis (17 x 10^9^/L), and his platelet count and hemoglobin concentration were normal. Abnormal lymphoid cells were also present in the bone marrow (Figure 1). The proportion of prolymphocytes and lymphocytes in the bone marrow were 2.8% and 67.2%, respectively. He immediately received chemotherapy consisting of cyclophosphamide, vincristine, doxorubicin, dexamethasone, and rituximab (R-hyper-CVAD) [10,11], resulting in complete remission. After finishing this initial treatment, it turned out that the majority of proliferated lymphoid cells in the samples taken before treatment were positive for CD5, CD23, CD38, B-cell lymphoma (BCL)-2, as well as CD20, and negative for CD3, CD10, CD56, Cyclin D1, Cyclin D2, TP53, and terminal deoxynucleotidyl transferase in immunohistochemistry and/or flow cytometry. Both IRF-4 and ZAP-70, but not TP53, were strongly expressed in the lymphoid cells (Figure 1), and approximately 10% of the cells were positive for Ki67. We then reconfirmed that there were few large cells in each sample of peripheral blood, bone marrow, and the biopsied lymph node. Based on these morphological findings and the immune phenotype, he was diagnosed with CLL without the Richter’s transformation. According to the diagnosis of CLL, clinical stage before the initial treatment was reestablished as the Binet B and Rai II. Cytogenetic analysis with the G-banding method without the addition of mitogen revealed a chromosomal translocation, 46,XY,t(1;6)(p34.1;p23) in 10 of 20 analyzed metaphase cells (Figure 2). No other abnormality was found with chromosomal analysis with G-banding, and del11q was not detected in FISH analysis. He received a total of 6 courses of R-hyper-CVAD and high-dose chemotherapy with ranimustine, cytarabine, etoposide, and melphalan, followed by autologous peripheral blood stem cell transplantation (auto-PBSCT). Since then, his CLL has not relapsed for 20 months.

## Discussion

Chromosomal translocations are rare but usually involve the *IG* genes in CLL [4]. However, our patient showed the reciprocal chromosomal translocation t (1;6)(p34.1;p23), which is not associated with the *IG* genes. So far, there have been a few reports on t (1;6) in CLL, including a study on 8 patients with the t(1;6)(p36 ~ 33;p25 ~ 23) found in the Belgian Cytogenetic Institutes database [5]. The 8 patients showed an advanced and progressive clinical course, with three of them being complicated with Richter’s transformation. In addition, most of these patients required conventional chemotherapy shortly after diagnosis. Our patient also showed rapid progression of the disease after the sudden onset. CLL cases with t (1;6)(p35;p25) in the complex karyotype have also been reported [7,8]. Thus, CLL with t (1;6) may be of a distinct type. On the other hand, there have been more CLL cases with t(1;6) who had different breakpoints such as 1q25, 1q21, 6p11 and 6p12 [9], but the features of these cases remain unknown.

The CLL cells in our case significantly expressed IRF4 in immunohistochemistry. CLL cells are known to frequently express IRF4 [12], which was also implicated in the pathogenesis of CLL with t(1;6) [5]. However, IRF4 expression in the patients with t(1;6) has not been shown in the previous reports. Overexpression of *IRF4* mRNA is rarely associated with a somatic mutation in the *IRF4* gene [13], but unfortunately, genetic analysis for *IRF4* was not performed in our case. Whether chromosomal rearrangement of t(1;6) is inducible to express *IRF4* as well as the somatic point mutation in the *IRF4* gene remains unclear.

Our case also showed robust expression of ZAP-70 (Figure 1), which is associated with poor prognosis and aggressive clinical course in CLL. The immunoglobulin variable heavy chain (*IgVH*) gene was not investigated in our case, as it is known that the expression of ZAP-70 is strongly correlated with the unmutated *IgVH* gene [14]. Interestingly, almost all CLL patients with t(1;6) exhibited the unmutated *IgHV* gene [5]. As for other predictors of poor prognosis, CD38, but not TP53 or Ki67, was significantly expressed in our case.

The most common treatment for CLL is chemotherapy with a purine analogue, although high-dose chemotherapy with auto-PBSCT may be another option if a patient responds to conventional chemotherapy [15]. In the previous report of CLL with t(1;6) by Michaux et al. [5], 2 of 8 patients also underwent high-dose chemotherapy with auto-PBSCT. Notably, both of them achieved complete remission and their disease did not relapse during observation. In contrast, of the 8 CLL patients with t(1;6), patients who received purine analogues were especially complicated with Richter’s transformation. Proceeding from these findings and good response to the initial treatment, our patient received a total of 6 courses of R-hyper-CVAD followed by high-dose chemotherapy with auto-PBSCT. However, owing to the limitation in the number of patients, appropriate treatment for CLL with t(1;6) remains to be determined.

In conclusion, we report the presence of CLL with t(1;6) in Asians as well as Europeans, suggesting that t(1;6) may define a distinct type of CLL. We should anticipate chromosomal abnormalities such as t(1;6) in CLL as the prognosis may be affected.

## Consent

Written informed consent was obtained from the patient for publication of this Case report and any accompanying images. A copy of the written consent is available for review by the Editor-in-Chief of this journal.

## Competing interests

The authors declare no conflict of interest.

## Authors’ contributions

KH, KI, KO, YT: wrote the manuscript, treated patients. HM, MF, HT, HO, HN: treated patients, analyzed data. KT and MA: performed histopathological analysis. All authors read and approved the final manuscript.
